# Ultrasound measurement of the size of the anterior tibial muscle group: the effect of exercise and leg dominance

**DOI:** 10.1186/1758-2555-3-18

**Published:** 2011-09-13

**Authors:** Karen McCreesh, Sinead Egan

**Affiliations:** 1 Dept of Physiotherapy, University of Limerick, Limerick, Ireland

## Abstract

**Background:**

Knowledge of normal muscle characteristics is crucial in planning rehabilitation programmes for injured athletes. There is a high incidence of ankle and anterior tibial symptoms in football players, however little is known about the effect of limb dominance on the anterior tibial muscle group (ATMG). The purpose of this study was to assess the effect of limb dominance and sports-specific activity on ATMG thickness in Gaelic footballers and non-football playing controls using ultrasound measurements, and to compare results from transverse and longitudinal scans.

**Methods:**

Bilateral ultrasound scans were taken to assess the ATMG size in 10 Gaelic footballers and 10 sedentary controls (age range 18-25 yrs), using a previously published protocol. Both transverse and longitudinal images were taken. Muscle thickness measurements were carried out blind to group and side of dominance, using the Image-J programme.

**Results:**

Muscle thickness on the dominant leg was significantly greater than the non-dominant leg in the footballers with a mean difference of 7.3%, while there was no significant dominance effect in the controls (p < 0.05). There was no significant difference between the measurements from transverse or longitudinal scans.

**Conclusions:**

A significant dominance effect exists in ATMG size in this group of Gaelic footballers, likely attributable to the kicking action involved in the sport. This should be taken into account when rehabilitating footballers with anterior tibial pathology. Ultrasound is a reliable tool to measure ATMG thickness, and measurement may be taken in transverse or longitudinal section.

## Background

Gaelic football is a high speed field sport played in Ireland and internationally. In common with other field sports involving kicking, physical contact and running, there is a high incidence of ankle and anterior tibial symptoms in Gaelic football players [1,2]. These symptoms may be associated with dysfunction of the muscles of the anterior compartment of the leg, located anterolaterally between the tibia and fibula, bounded deeply by the interosseous membrane and superficially by the deep fascia of the leg. The muscles in the anterior tibial muscle group (ATMG) include tibialis anterior, extensor hallucis longus, and extensor digitorum longus. As well as kicking from the hand and the ground, Gaelic football also involves an additional kicking skill, called "soloing". In order to travel with the ball, for every four steps taken the player must either bounce the ball or drop it onto the foot, kicking it back to his or her own hand-this is a 'solo', and would be likely to involve significant ATMG activity in dorsiflexing the foot.

Knowledge of normal muscle characteristics is crucial in planning rehabilitation programmes for patients with muscle injury, as reduction in muscle size has been reported in injured populations [3,4]. Real-time ultrasound has been shown to provide both a reliable [5] and valid measure of skeletal muscle size when compared to gold standards such as MRI and CT [6]. Specifically in the ATMG, Martinson & Stokes [7] found ultrasound to be a reliable method of muscle size measurement, and that cross-sectional area is correlated with thickness measurements. These authors took the scans in transverse section, as this is the appropriate view for CSA measurements, and measured muscle thickness also in this plane. In longitudinal section, muscle fascicles, and fascial and bony interfaces are viewed along their length. In transverse section, they are viewed in cross-section. In an area such as the lower leg, which is convex in shape, aligning the probe in longitudinal section may allow better congruency to the tissue and thus better image quality. However it may be easier to define the area of thickness measurement in transverse section, as due to the circular shape of the muscle, the area of maximum thickness can be easily determined. No studies could be found where muscle thickness measurement in the two planes has been compared in humans. A study comparing scans in the two planes would provide information as to whether one scanning direction has superior reliability or ease of use, and thus make it the recommended direction to use.

OSullivan et al [8] demonstrated a positive effect of limb dominance on muscle size and strength in the hamstrings of Gaelic footballers, however little is known about the effect of limb dominance and sport-specific skills on the dimensions of the anterior tibial muscle group (ATMG). Kelly and Stokes [9] examined cross-sectional area of the ATMG in healthy females by ultrasound scanning, and noted a 7.2% side-to-side difference, however it was not stated whether this difference favoured the dominant limb, or whether there might have been any activity or sport-specific hypertrophy in the subjects.

### Aim

The aims of this study are to assess the effect of limb dominance and sports specific activity on ATMG thickness in Gaelic footballers and non-football playing controls using ultrasound measurements, and secondarily to compare results from transverse and longitudinal scans.

## Methods

### Subjects

A convenience sample of 20 healthy subjects (10 college level footballers and 10 non-active controls) was recruited from a University student population. Information leaflets were provided to volunteers, and subjects were selected based on the inclusion and exclusion criteria below. To be included, footballing subjects had to be over 18 years, be an out-field member of a college-level football team, be playing football for a minimum of 5 years, and have no current or recent (3 month) history of lower limb injury. Goalkeepers were excluded as they would not perform the same amount of kicking as outfield players. Recently injured players were excluded, as recovery may not have been complete. Control subjects were required to be 18 years, and to not engage in any sports or activities that may affect lower limb muscle strength. Written informed consent was obtained from each participant and ethical approval was obtained from the University Research Ethics Committee.

### Procedure

Before the testing procedure began, subject characteristics were recorded. These were age, gender, height, weight, leg dominance, frequency of training per week, position of play on the field. Leg dominance was subjectively reported by the footballers, and determined in controls, by asking them to kick a ball that was placed centrally in front of them.

**Figure 1:**
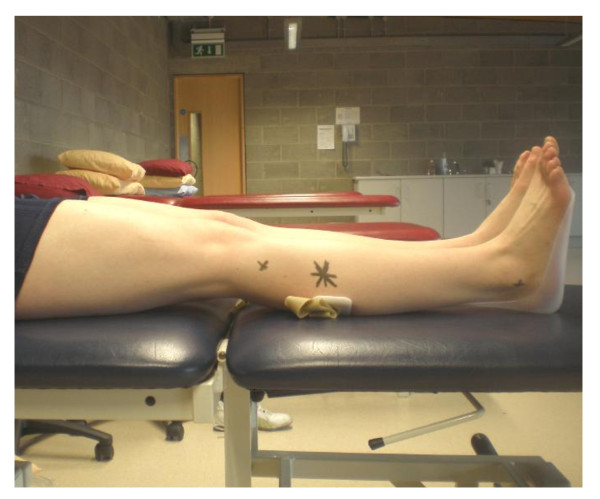
**Scanning position**. Test position with star indicating point of measurement. The lateral malleolus and head of fibula can also be seen marked with an 'x'.

A Esaote Aquila ultrasound scanner (Pie Medical, Maastricht, The Netherlands) with an 8 MHz linear array transducer (Esaote Europe, The Netherlands; 40 mm footprint) was used for measuring both the left and right anterior tibial muscle groups. Measurements were taken in both transverse and longitudinal directions. The testing positioning and procedure used was based on previous research measuring anterior tibial muscle size using real-time ultrasound imaging [7]. Subjects were positioned in long sitting on a plinth, with the hip in 90 degrees flexion, the knee extended and the ankle resting in zero degrees in an ankle foot orthosis (AFO). The subjects wore shorts in order to leave the lower limbs exposed. Scans were taken at a point 20% of the distance from the head of fibula to the tip of the lateral malleolus. The distance was measured using a measuring tape. This point was then marked on the subject's leg *(*Figure 1).

Ultrasound coupling gel was used over the skin and transducer head. The transducer was held such that the ultrasound beams were aimed at 90 degrees to the muscle borders, by visually ensuring the transducer was held perpendicular to the skin, to ensure a clear image. Controls for contrast, brightness and near and far gain were adjusted to obtain a satisfactory image. Care was taken not to deform the underlying tissues and distort the images by using the minimum pressure required to create a clear image. Transverse measurements were taken by placing the probe perpendicular to the direction of the anterior tibial muscles. Longitudinal measurements were then taken by aligning the probe along the direction of the muscles. Three measurements were taken in each direction in order to reduce error, removing the probe between trials. Linear dimensions of the anterior tibial muscle group have previously been shown to reflect CSA [7]. Scans were taken by SE, a novice researcher, however one-to-one training in scanning, and full supervision, was given by a researcher trained and experienced in musculoskeletal sonography.

**Figure 2:**
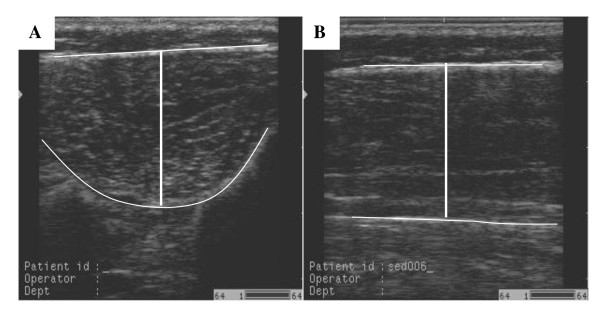
**Ultrasound images of anterior tibial muscle group, showing line of thickness measurement**. A: Transverse scan. B: Longitudinal scan

The images were stored and transferred to a computer for measurement. Measurement of muscle thickness was carried out using Image J software (National Institute for Health, Bethesda, MD, USA). Each image was measured by two examiners. Measurement of anterior to posterior thickness involved placing a vertical line spanning the widest part of the echogenic tissue interface between the muscle belly and the muscle fascia, as seen in Figure 2. Three images were measured for each limb and scanning direction, and the mean thickness from these three images used for analysis. Measurement of the scans was undertaken blind to subject details (i.e. group or leg dominance).

### Statistical Analysis

SPSS Version 15.0 software was used for all statistical analysis. All data was found to be normally distributed (Kolmogorov-Smirnov *p >*0.05). Personal data (age, height, weight) was compared between groups using independent t-tests. Intraclass correlation coefficients (ICCs) were obtained for inter-tester reliability of the measurement of the scans by the 2 examiners, using the mean thickness obtained by each examiner. Transverse and longitudinal measurements were compared within subjects using paired t-tests, and the association between measures was assessed with Pearsons correlation. A Two-way ANOVA (leg × subject group) was used to examine differences in ATMG thickness between the dominant and non-dominant legs according to group. Significance level was set at p < 0.05.

## Results

### Subjects

20 subjects were recruited (10 footballers and 10 controls). Mean age was 21.3 years (range 18-25), and all were right leg dominant. The mean weight and height of controls was 67.1 kg and 172.6 cm respectively, while the mean weight and height of footballers was 72.5 kg and 172.6 cm respectively. Each group contained 5 males and 5 females. There was no significant difference between the groups on any of these variables (p > 0.05), apart from sport-playing history.

### Reliability and comparison of scan direction

**Figure 3:**
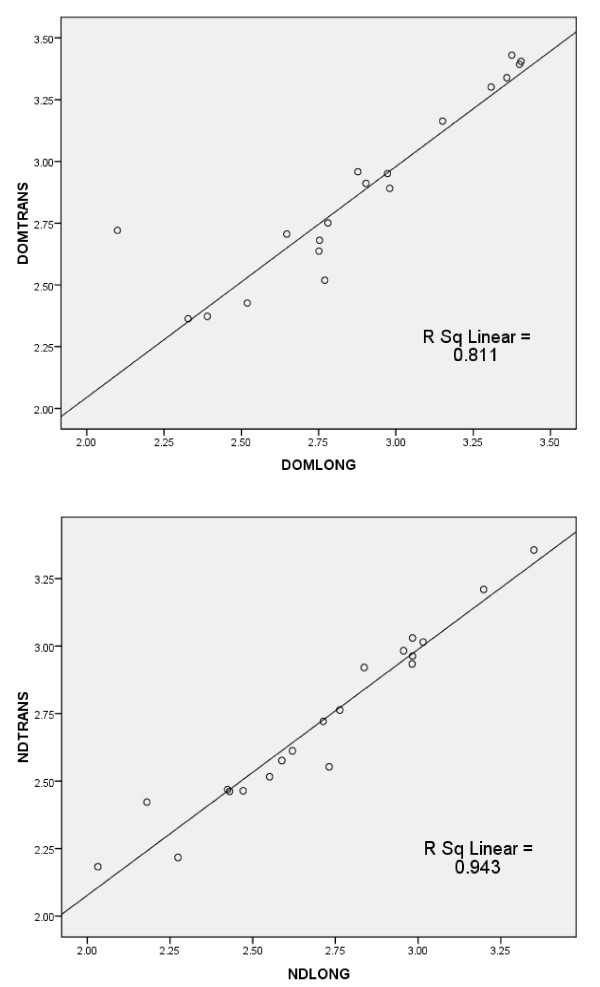
**Pearson correlation graphs showing close association between transverse and longitudinal measurements (cm)**. NDTRANS = Non-dominant transverse measurement; NDLONG = non-dominant longitudinal measurement; DOMTRANS = dominant transverse measurement; DOMLONG = dominant longitudinal measurement.

Intra-class co-efficients for the inter-rater reliability of measurement of the scans by 2 examiners demonstrated excellent reliability with values of 0.997 (95% confidence interval (CI) = 0.986-0.998) and 0.993 (95% CI = 0.984-0.997) for transverse measurements on the dominant and non-dominant sides respectively, and 0.994 (95%CI = 0.985-0.998) and 0.992 (95%CI = 0.981-0.997) for longitudinal measurements on the dominant and non-dominant sides respectively. There was no significant difference between measurements taken using the transverse and longitudinal approaches for either dominant (p = 0.793) or non-dominant legs (p = 0.442), therefore these measurements were collapsed into a single measure obtaining a mean muscle group thickness for each leg of each subject. Further evidence of the close association between measurements in transverse and longitudinal directions can be see in Figure 3, which illustrates the correlation between measurements in the two planes for non-dominant and dominant ATMG thicknesses (Non-dominant thickness r = 0943; dominant thickness r = 0.811).

### Dominance and Group Comparison

Mean ATMG thickness (± standard deviation) results can be see in Table 1.

**Table 1:** Mean (± SD) Anterior Tibial Muscle Group Thickness (cm)

	Football players	Controls
Dominant limb	3.052 (± 0.379)	2.659 (± 0.336)

Non-dominant limb	2.842 (± 0.373)	2.605 (± 0.257)

Between-limb Difference	0.21*	0.054

* indicates a statistically significant difference

The results of the two-way ANOVA revealed a significant interaction for the effects of group and leg on ATMG size, showing that the effect of leg dominance differed between the footballers and controls (F _3,39 _= 8.18; p = 0.007). Examining the data shows that the dominant ATMG was larger only in the footballers, and not in controls. A mean between-leg difference of 0.21 cm was found in the footballers, which can be expressed as the dominant muscle group being 7.3% greater in thickness than the non-dominant muscle group.

## Discussion

The results found in this study highlight some important aspects for consideration in measurement of muscle size in athletes. Inter-rater reliability of scan measurement was high, similar to previous research using ultrasound, which reported high reliability for both linear and CSA measurements between scans and days [5]. Measurements from longitudinal and transverse images were not significantly different, and were equally reliable to measure; therefore either method may be used. Longitudinal measurements may allow multiple areas of thickness to be measured on a single image which may be more appropriate for studies assessing change over time, while also providing greater ease of transducer alignment on the lower leg, therefore this would be our method of choice.

Secondly, this study found that a small, but statistically significant, difference exists in the size of the anterior tibial muscle group in dominant, compared with non-dominant, legs in Gaelic Footballers, representing a 7.3% increase in thickness on the dominant side. This significant side-to-side difference was not seen in age-matched, non-football playing controls and therefore, the difference seen in footballers can be attributed to sports specific performance. This is consistent with previous research on soccer players which found a similar dominance effect in the medial gastrocnemius muscles, where a side-to-side difference of 0.18 cm, equating to an overall difference of 6%, was found [10]. Similarly the study by O'Sullivan et al [8] of muscle size in Gaelic Footballers found a significant dominance effect in hamstrings (representing a 6.5% difference), but no such difference in quadriceps muscles. When comparing with the Kelly and Stokes study [9] of the ATMG, a similar side-to-side difference has been identified, however no association was made in that study with side of dominance or sport-related hypertrophy. There was no dominance effect on muscle size in the control group in this study. This finding is relevant for the clinician in terms of gauging recovery in the dominant limb post-injury. The use of ultrasound scanning in rehabilitative settings is increasing [11] and for clinicians with the relevant training, the measurement of muscle dimensions could form a useful outcome assessment for patients undergoing post-injury rehabilitation.

Footballers also had a significantly larger muscle thickness on their dominant leg, compared to the dominant legs of controls, however no such difference existed in comparison of non-dominant legs between groups. This indicates training-induced hypertrophy of the dominant limb in the footballers. Training, and in particular, resistance training, has been shown to result in altered muscle architecture and muscle producing force [12] and hence, the sports-specific actions of Gaelic football (kicking and soloing) may be likened to a strengthening exercise for the ATMG. Martinson and Stokes [7] concluded that the potential of linear dimensions in assessing changes in muscle size with atrophy and hypertrophy has yet to be established. This study provides some evidence that linear dimensions can detect muscle hypertrophy in the dominant limb of active subjects. However, it is not known whether such a measure can detect longitudinal changes in muscle dimensions.

Muscle atrophy following injury has traditionally been measured using measures of limb girth, with atrophy defined as a greater than one centimetre difference between limbs [13]. However, this has proved inaccurate and often grossly underestimates muscle wasting, as it includes a measure of the subcutaneous tissue and fat. Ultrasound imaging is a quick, easily available, reliable alternative [6]. The measurement protocol in this study could be reliably used to examine muscle size changes in athletes with ankle injury to determine whether atrophy occurs in the ATMG, with an appropriate correction for the size of the dominance effect. A longitudinal study examining subjects over a given period would give more information on the time course of the changes to the muscle as a direct result of training, or of injury. Such a study would also be beneficial in the case of injury, to quantify the extent of weakness and the rapidity with which it occurs and so plan an appropriate rehabilitation. Previous studies have found selective muscle hypertrophy, for example within the quadriceps muscle group [14], and therefore a more detailed assessment of the individual muscles within the anterior tibial muscle group may be warranted in order to gain more information regarding structural changes.

The relationship between muscle size and strength was not assessed in this study. However it would be important to assess the relationship between the size and morphology of this muscle group and muscle performance. The anterior tibial muscle has a fibre type composition ranging from 65% up to 90% type 1 fibres [15], which may change under various conditions. Although muscle force and CSA are strongly and directly related in studies of healthy muscle, muscle volume appears to be better related to muscle torque [16]. Therefore the calculation of muscle volume may allow for a more accurate prediction of the contribution of atrophy to muscle weakness [17].

Limitations to this research include small sample size, convenience sample, and possibility of measurement error. Error in scan measurement was reduced by the use of two examiners in measuring each of the scans, and the provision of close training and supervision by a researcher experienced in rehabilitative ultrasound scanning. A sample of convenience was used, however the sample recruited could be considered representative of the Gaelic football playing population. In addition, only one part of the muscle group was measured, and the results may not reflect those that would be obtained using other scanning sites.

## Conclusions

In conclusion, a side-to-side difference, favouring the dominant leg, exists in the size of the anterior tibial muscle group in Gaelic footballers. This difference is not seen in inactive, age-matched controls and so is likely to be attributable to the action of kicking in the sport. The clinician may take this difference into consideration when treating football players with a lower limb or ankle injury, by using ultrasound scanning to monitor change in muscle dimensions. Future research could examine this dominance effect in other sports and muscle groups, as well as the association between muscle size and strength or performance.

## Competing interests

The authors declare that they have no competing interests.

## Authors' contributions

KM was involved in the design of the study, data collection, data analysis, and drafting of the manuscript. SE was involved in the literature review, design of the study, recruitment, data collection, data analysis and drafting of the manuscript. All authors read and approved the final manuscript.
